# Anastrozole (‘Arimidex’) blocks oestrogen synthesis both peripherally and within the breast in postmenopausal women with large operable breast cancer

**DOI:** 10.1038/sj.bjc.6600587

**Published:** 2002-10-21

**Authors:** W R Miller, M Stuart, T Sahmoud, J M Dixon

**Affiliations:** Breast Unit, Western General Hospital, Edinburgh EH4 2XU, Scotland, UK; AstraZeneca, Alderley Park, Cheshire, UK

**Keywords:** postmenopausal breast cancer, anastrozole, aromatase inhibitor, tumour, oestrogens, neoadjuvant therapy

## Abstract

The effect of anastrozole on peripheral and tumour aromatase activity and oestrogen levels in postmenopausal patients with oestrogen receptor-rich breast tumours was investigated. Twenty-six patients were randomly allocated to treatment with anastrozole 1 mg (*n*=13) or 10 mg (*n*=13), once daily. Before and after 12 weeks' treatment, patients were infused with ^3^H-Δ_4_ androstenedione (20 MBq) and ^14^C-oestrone (E_1_) (1 MBq) for 18 h. Oestrogens were purified from excised tumours and plasma samples taken after each infusion. Peripheral and tumour aromatase activity and tumour E_1_ uptake were calculated from levels of ^3^H and^ 14^C in purified E_1_ fractions from tumour and plasma. Endogenous tumour oestrogens were measured by radioimmunoassay. Twenty-three patients were available for analysis (1 mg group, *n*=12; 10 mg group, *n*=11). Following treatment, anastrozole (1 and 10 mg) markedly inhibited peripheral aromatase in all patients (the difference between pre- and on-treatment values being highly significant *P*<0.0001). *In situ* aromatase activity was also profoundly decreased by anastrozole treatment in 16 of 19 tumours (the difference with treatment also being highly significant *P*=0.0009). Most tumours were able to concentrate E_1_ beyond levels in the circulation; anastrozole treatment had no consistent effect on uptake of E_1_. Endogenous tumour levels of both E_1_ and oestradiol (E_2_) were significantly reduced with therapy (*P*=0.028 for E_1_ and *P*=0.0019 for E_2_). Anastrozole (1 and 10 mg daily) effectively suppresses aromatase activity, and subsequently oestrogen levels, within the breast tissue of postmenopausal women with large or locally advanced, operable, oestrogen receptor-rich breast cancers.

*British Journal of Cancer* (2002) **87**, 950–955. doi:10.1038/sj.bjc.6600587 www.bjcancer.com

© 2002 Cancer Research UK

## 

In approximately one-third of cases of advanced breast cancer, oestrogen deprivation causes tumour regression (Jackson and Lowery, 1987). This effect is associated with both anti-oestrogens that block oestrogen receptors (e.g. tamoxifen) and inhibitors of oestrogen biosynthesis. The latter may be most specifically achieved by targeting the aromatase enzyme, which catalyses the final step in the oestrogen biosynthetic pathway (Miller, 1996).

There are two major types of aromatase inhibitors (AIs): Type I inhibitors are androgens or androgen analogues that block the substrate binding site on the enzyme; Type II inhibitors are non-steroidal agents that interfere with the cytochrome P450 moiety in the aromatase molecule (Miller, 1996). Although the agents inhibit peripheral aromatase activity, the relationship between potency in lowering plasma oestrogen levels and clinical effectiveness in treating breast cancer is poorly defined and it has been suggested that effects on circulating oestrogens do not entirely account for anti-tumour influences (Bajetta *et al*, 1999). Levels of oestrogens in the breast tissue (including tumours) of postmenopausal women may be higher than those in the circulation, and this may result from both increased local synthesis and/or active uptake from the circulation (Miller, 1996). It is therefore of interest to investigate the effect that AIs have on aromatase activity and oestrogen levels in breast tissue.

Anastrozole is a potent, orally active, selective, non-steroidal AI that markedly reduces the levels of circulating oestrogens in postmenopausal women with breast cancer (Plourde *et al*, 1995; Geisler *et al*, 1996). As a second-line agent for adjuvant therapy in postmenopausal women with advanced breast cancer, anastrozole has been shown to offer significant clinical benefits when compared with the progestin, megestrol acetate (MA) (Buzdar *et al*, 1996, 1998). Anastrozole shows significant benefit over tamoxifen with respect to TTP (Bonneterre *et al*, 2000; Buzdar *et al*, 2000) when given as first-line treatment of postmenopausal women with advanced hormone-sensitive breast cancer. Finally, anastrozole is highly effective in reducing tumour volume in the neoadjuvant therapy of breast cancer in postmenopausal women (Dixon *et al*, 2000). Indeed, 16 out of 18 patients in this trial who were originally registered for a mastectomy at the trial onset, were suitable for breast conservation following anastrozole treatment. However, the effect of anastrozole on tumour aromatase has not been reported to date.

## MATERIALS AND METHODS

### Patients and treatments

The eligibility criteria for patients participating in this study have been described previously (Dixon *et al*, 2000). In brief, subjects were postmenopausal women with oestrogen-receptor (ER)-rich breast tumours (⩾20 fmol mol^−1^ protein). All breast tumours were operable and either larger than 3 cm (T_2_ [>3 cm], T_3_, N_0–1_, M_0_), or locally advanced (T_4b_, N_0–1_, M_0_).

Twenty-six patients were recruited into a randomized, double-blind, single-centre study in which they received anastrozole either 1 or 10 mg orally once daily for 12 weeks, to be followed by definitive surgery. The random scheme and associated sealed envelopes were produced by computer software that incorporates a standard procedure for generating random numbers.

### Infusion of radiolabelled steroids

Before initiation of anastrozole, each patient was infused with 20 MBq ^3^H-Δ_4_ androstenedione and 1 MBq ^14^C-oestrone (E_1_) in 50 ml of 95% plasma protein solution and 5% ethanol. The infusion was given as an initial 10 ml bolus followed by an 18-h infusion at 2 ml h^−1^. Delivery was by a 50 ml syringe attached to a Teflon-coated tubing and transmitted through a 21-gauge venflon into a peripheral vein. The infusion was immediately followed by an open wedge biopsy to sample the tumour. Peripheral blood (40 ml) and residual infusion fluid were also taken for analysis.

The process was repeated after 12 weeks of anastrozole therapy, with breast tissue being removed during definitive surgery at this time.

### Extraction and purification of radiolabelled steroids

The method used to extract and purify radiolabelled oestrogens from tissue samples was essentially that described by other workers (James *et al*, 1987; Reed *et al*, 1989). Tissue (0.4–0.6 g) was finely sliced and pulverised in liquid nitrogen using a microdismembrator (Braun). The resultant powder was vortexed with radioinert E_1_ (500 μg in alcohol) in distilled water and left at room temperature for 30 min. The extract was then added to ethanol : acetone (1 : 1) and left at room temperature for 30 min before centrifugation. The supernatant was decanted and the pellet resuspended in ethanol : acetone (1 : 1) and recentrifuged. The resultant supernatant was pooled with the first sample and evaporated down to the aqueous residue, to which methanol (70% in water) was added and left overnight at –20°C. The mixture was then centrifuged and the supernatant evaporated down to the aqueous residue, which was partitioned between acetate buffer (0.1 M, pH 4.5) and diethylether. Ether extracts were dried, reconstituted in hexane : chloroform (80 : 20) and resolved by chromatography on Lipidex using serial elution with hexane : chloroform (80 : 20, 50 : 50, 40 : 60). Fractions containing E_1_ (hexane : chloroform [50 : 50]) were evaporated to dryness and reconstituted in ethanol for further purification using thin layer chromatography for 1 h on silica gel plates using cyclohexane : ethyl acetate (55 : 45). The E_1_ fraction was eluted and read at 280 nm on a spectrophotometer to monitor recovery. The radioactivity in the fraction was then measured (in disintegrations per min, d.p.m.) using a scintillation counter. Plasma samples (10 ml) were extracted with diethylether, reconstituted in 70% methanol and further processed as above.

Each sample was counted for 5×50 min, together with vials containing known levels of ^3^H and ^14^C alone and in combination and representing the range of absolute d.p.m. and ratio of isotopes encountered in the study. Control vials were also counted comprising ‘blank’ areas from the plates and plasma/tumour extracts from patients not receiving radioisotopes. Additionally, random samples were cross-checked on a separate scintillation counter designed and set up for low counts as in ^14^C dating. The cross-over of ^14^C in the ^3^H channel was −0.4% to 0.1% and no ^3^H was counted in the ^14^C channel.

Random samples were also subject to chemical derivative formation and shown to have consistent specific radioactivity throughout the procedure, thus confirming purity of oestrone fractions (Miller and Forrest, 1976).

**Figure 1:**
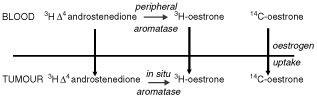
Tumour uptake and *in situ* synthesis of oestrogens as estimated by measurement of radioactivity.

Tumour uptake of E_1_ was calculated as the ratio of the level of ^14^C-E_1_ in the tumour to that in the plasma following its infusion. Aromatase activity was measured by the synthesis of ^3^H-E_1_ from the infused precursor ^3^H-Δ_4_ androstenedione. Plasma ^3^H-E_1_ synthesis therefore represents peripheral aromatase activity. However, tumour aromatase activity cannot simply be taken as tumour ^3^H-E_1_ synthesis, since it is likely that some of the ^3^H-E_1_ found in the breast is formed peripherally, secreted into the circulation and then taken up by breast tissue. A correction factor is derived from the level of ^14^C-E_1_ taken up by the tumour, since any ^3^H-E_1_ present in the breast in excess of that expected from uptake represents tumour E_1_ synthesis (Figure 1

).

Therefore:

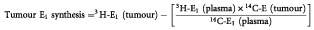


### Radioimmunoassay of endogenous oestrogens

Tissue samples (approximately 0.4–0.6 g) were extracted as for radiolabelled oestrogens, apart from the addition of tracer oestrogens in place of radioinert steroid. Extracts were then reconstituted in toluene : methanol (92 : 8), and subjected to chromatography on Sephadex LH-20. Fractions containing either E_1_ or oestradiol (E_2_) were then evaporated to dryness and analysed by radioimmunoassay as described previously (Thijssen *et al*, 1991; Miller *et al*, 1998). Level of detection was 40 fmol g^−1^ for oestradiol and 62.5 fmol g^−1^ for oestrone as calculated from the blanks in the 12 assays performed for sample measurements and a 99% probability of the value being by chance (Koch and Peters, 1996). Correction for ^14^C labelled oestrogen in the purified fraction for E_1_ (median 0.32 pmol) ranged from 0.06 to 0.98 pmol and for E_2_ 0.00 to 0.61 pmol (median 0.18 pmol).

### Statistical methods

Differences in peripheral and tumour aromatase activity and tumour endogenous oestrogen levels between the two treatment doses (1 and 10 mg) were analysed using analysis of covariance (ANCOVA), including terms for pre-treatment and treatment measurements. Differences between assessments (before treatment and after surgery) were analysed using a paired *t*-test. Data for the peripheral and tumour aromatase activity are expressed as d.p.m. ^3^H-E_1_ g^−1^. Endogenous E_2_ and E_1_ levels in breast tumour are expressed as pmol g^−1^. Oestrone uptake was expressed as the ratio of tumour : plasma ^14^C-E_1_.

## RESULTS

### Patient characteristics

Twenty-four out of the 26 patients recruited into the study were eligible for analysis. Two patients (one in each group) were found to have ER-negative tumours and so were excluded from the efficacy analysis. One further patient (from the 10 mg group) withdrew from the study because of adverse events (headaches, depression, and tiredness). Thus, the data from 23 patients were available for analysis. The demographic and baseline tumour characteristics of the remaining patients were comparable between the two groups (Table 1

**Table 1:**
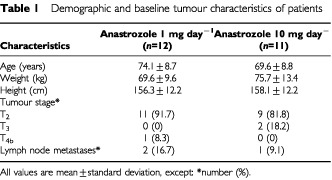
Demographic and baseline tumour characteristics of patients

). The histological tumour volume was a median 1.9 cm^3^ (range 0.1–6.6 cm^3^) for the anastrozole 1 mg group and 1.9 cm^3^ (range 0.1–7.5 cm^3^) for the anastrozole 10 mg group.

All patients gave their written informed consent, and appropriate ethics committee approval was obtained prior to initiation of the study.

### Peripheral aromatase activity

**Figure 2:**
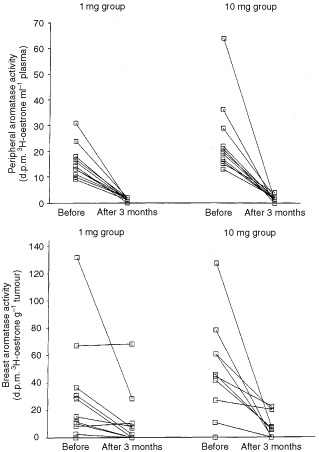
Effect of neoadjuvant anastrozole on peripheral aromatase activity (upper panel) and aromatase activity within the breast (lower panel) at baseline and after 3 months' treatment.

Before treatment, there was evidence for peripheral aromatase activity in all 23 evaluable patients (Figure 2

). In terms of percentage conversion, these values ranged from 0.39 to 1.91%. Following 12 weeks' anastrozole treatment (1 and 10 mg) there was a profound decrease in aromatase activity in all patients, irrespective of dose. Indeed, in five cases, no ^3^H was detected in E_1_ fractions from the plasma of treated patients (range of ^3^H 0–7 d.p.m., ^14^C 44–156 d.p.m.). Percentage conversion on treatment ranged from 0 to 0.18%. The decrease in peripheral aromatase therapy was highly significant (*P*<0.0001) and the median value for inhibition was 94%. There were no significant differences between the two treatment doses (*P*=0.8793).

### Tumour aromatase activity

Before treatment, aromatase activity was detected within the tumour in 19 of 23 patients and the remaining four tumours had no evidence for aromatase activity. After 3 months of anastrozole therapy, 17 tumours exhibited a fall in activity (Figure 2), two (from the 1 mg group) showed increased *in situ* aromatase, and aromatase activity remained undetectable in four tumours. (The d.p.m. in the oestrone fractions counted from on-treatment patients were ^3^H –1 to 5 (apart from a single tumour with a value of 13) and ^14^C 15 to 40.) The difference between pre-treatment and treated specimens was statistically significant (*P*=0.0009) and the median value for inhibition was 89% (although it should be noted that other factors such as the further metabolism of oestrogen may compromise exact quantitation) (Larionov *et al*, 2002). There were no significant differences between the two treatment doses (*P*=0.34).

### Tumour uptake of oestrone

**Figure 3:**
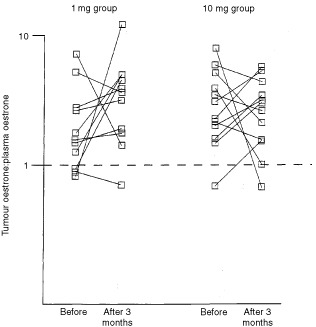
Effect of neoadjuvant anastrozole on oestrone uptake, as measured by the ratio of tumour to plasma oestrone at baseline and after 3 months' treatment.

The uptake of E_1_ into the tumour as measured by the ratio of ^14^C-E_1_ in the tumour to that in plasma is shown in Figure 3

. Values were typically >1 indicating the potential of tumour tissue to concentrate E_1_ from the plasma against a concentration gradient. Whilst treatment with anastrozole could be associated with changes in uptake, the direction of effect was not consistent, and the difference between pre-treatment and treatment values was not statistically significant (*P*=0.53).

### Tumour endogenous oestrogen levels

**Figure 4:**
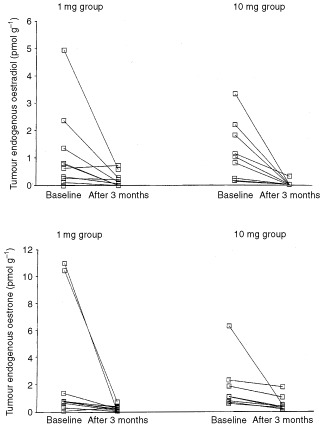
Effect of neoadjuvant anastrozole on the endogenous oestradiol (upper panel) levels within the breast at baseline and after 3 months' treatment and oestrone (lower panel) levels within the breast at baseline and after 3 months' treatment.

The effects of treatment on tumour levels of E_2_ and E_1_ are shown in Figure 4

. In the majority of cases, values fell markedly with therapy (indeed in five tumours levels of oestradiol were undetectable after treatment) such that the levels were statistically lower in treated specimens (*P*=0.028 for E_1_ and *P*=0.0019 for E_2_). In two tumours, it was not possible to observe a fall in oestradiol following treatment (in one of these tumours oestrone also did not decrease). There were no significant differences between the two treatment doses (*P*=0.234).

## DISCUSSION

Levels of oestrogens in postmenopausal breast tissue are higher than those in the circulation, this being at least partly due to local synthesis (Miller, 1996). Thus, to achieve optimal benefit in postmenopausal patients with breast cancer it may be necessary for an AI to block local aromatase activity within breast tumours as well as peripheral aromatase. Indeed, there is some evidence to suggest that tumours with aromatase activity are more likely to respond to inhibitors than those without detectable aromatase (Miller and O'Neill, 1987; Miller and Dixon, 2001).

The present study has demonstrated that 3 months of neoadjuvant anastrozole therapy in women with postmenopausal ER-rich breast cancer markedly suppressed peripheral aromatase activity in all patients. Similarly, treatment with anastrozole was associated with a clear decrease in activity in 16 of 19 tumours that demonstrated *in situ* aromatase activity pre-treatment. It is worth considering the reason for the inability to detect a clear reduction in activity in certain tumours. The fact that, in each of the patients, peripheral aromatase was profoundly influenced by treatment excludes the possibility that either the women were not compliant to taking the drug or that aromatase activity was in general resistant to anastrozole. The lack of effect would seem therefore either to be artefactual or specific to the local environment of the breast. In these respects, it is worthy of note that a decrease in activity was lacking in the paired non-malignant breast sample (from the single patient in whom material was available), whereas the expected inhibition was seen in all non-malignant samples from patients whose tumours were also affected (data not shown). This would seem to exclude the possibility that the tumours differentially possessed mutant aromatase which was resistant to the inhibitor (although such phenotypes have been constructed experimentally (Kadohama *et al*, 1992). The most likely explanation for the results is that, at the level of the breast insufficient drug accumulated to inhibit aromatase activity locally. Whilst this hypothesis is testable by measuring drug levels within the breast, these were not performed in the present study. Because aromatase activity is assessed by measuring ^3^H oestrone within the tissues, it is possible that the assessments are compromised by the presence of ^3^H oestrone remaining from the initial perfusion performed before administration of drug. Whilst this cannot be totally excluded (no tissue was collected immediately before the second perfusion) no radioactive oestrogen was detected in plasma taken either 14 days after the initial infusion or immediately before the second infusion; furthermore in a single patient from a separate study who declined a second perfusion, no radioactivity was detected in oestrogen fractions derived from breast tissue (data not shown).

Therapy was also associated with a marked reduction in tumour levels of endogenous oestrogens. Although the study was primarily set up to measure effects on *in situ* aromatase within the breast and not designed to determine quantitative changes in endogenous oestrogens (which would be complicated by the infusion of exogenous oestrogen), it was of interest to note that the median decrease in tumour E_2_ was 67% and in E_1_ was 70%. These figures are similar to those of another study involving postmenopausal patients (Geisler *et al*, 1999) in which 12 weeks of neoadjuvant therapy with anastrozole 1 mg daily, was associated with a geometric mean decrease in tumour levels E_2_ of 89% and E_1_ of 73%. These two studies demonstrate the potent inhibitory effect of anastrozole on tumour aromatase resulting in decreased *in situ* oestrogen synthesis.

Whilst consistent effects of anastrozole were noted in tumour aromatase activity and endogenous oestrogens, a more variable influence on tumour oestrogen uptake was apparent. Thus, whilst in general all tumours displayed the ability to concentrate oestrone from the circulation both before and during treatment, occasionally therapy was associated with marked changes. The possibility that these reflect methodological artefact cannot be completely excluded since replicate measurements were not possible on account of the limited size of the sample; however, replicate samples from other patients not on drug treatment never showed this degree of variation. It seems likely that the effects are a consequence of the changes in endogenous oestrogens following treatment with anastrozole and the different direction of effects reflect the differing mechanisms by which tumours may sequester oestrogen from the circulation.

The present study was also able to illustrate the clinical benefit of inhibiting aromatase activity as monitored by the marked reduction (>50%) in tumour volume (assessed by ultrasound) in 18 of the 24 patients (Dixon *et al*, 2000). It should be noted that changes in tumour aromatase following treatment did not always correspond with clinical behaviour. Thus, two of the three tumours in which anastrozole did not markedly reduce *in situ* activity, nevertheless responded to the drug. Presumably, the marked inhibitory effects on peripheral aromatase were sufficient to reduce tumour oestrogens and cause tumour response. Similarly, responses in tumours without aromatase activity are probably a consequence of inhibitory effects in other peripheral tissues. It is also of importance that as a result of anastrozole treatment in this study, only two patients required a total mastectomy. The remaining 16 of the 18 patients who were originally registered for a mastectomy only required wide local excision of the tumour.

The endocrinological effects of neoadjuvant anastrozole in the present report are entirely consistent with its anti-oestrogenic and anti-proliferative effects on tumour pathology, which have been reported previously in this group of women (Anderson *et al*, 2000), and are also consistent with the clinical efficacy of the compound in the treatment of advanced breast cancer in postmenopausal women (Buzdar *et al*, 1998; Bonneterre *et al*, 2000; Nabholtz *et al*, 2000). The results provided no evidence of any difference in the therapeutic effects of the two doses of anastrozole (1 and 10 mg) indicating that the currently recommended clinical dose of 1 mg daily is appropriate for obtaining adequate suppression of oestrogen levels within the breast.

It is of interest to compare the results of the present analysis with investigations into the effects of two other newer, potent, selective non-steroidal AIs (vorozole and letrozole) on breast tumour aromatase activity. In one study, eight previously untreated postmenopausal women awaiting mastectomy for breast cancer were treated with vorozole 2.5 mg daily. After 7 days, median *in vitro* tumour aromatase activity was 89% lower than in matched, control tumour specimens; similarly, median tumour levels of E_1_ and E_2_ in treated patients were 64 and 80% lower than in controls, respectively (de Jong *et al*, 1997). In another study that employed identical design and analytical methods to those reported here, similar levels of decreased tumour aromatase activity and tumour oestrogen concentrations were found following the use of neoadjuvant letrozole 2.5 mg daily for 3 months (Miller *et al*, 1998). Therefore, anastrozole, vorozole, and letrozole appear to have similar effects on tumour aromatase activity and tumour oestrogen levels in women with postmenopausal breast cancer.

In conclusion, anastrozole (1 or 10 mg daily) effectively suppresses aromatase activity, and thereby oestrogen levels, within the breast of postmenopausal women with large or locally advanced, operable, ER-rich breast cancers.
